# Generation of gene disruptions by transcription activator-like effector nucleases (TALENs) in *Xenopus tropicalis* embryos

**DOI:** 10.1186/2045-3701-3-21

**Published:** 2013-05-10

**Authors:** Yong Lei, Xiaogang Guo, Yi Deng, Yonglong Chen, Hui Zhao

**Affiliations:** 1 School of Biomedical Sciences, Faculty of Medicine, The Chinese University of Hong Kong, Shatin, New Territories, Hong Kong, P. R. China; 2 Key Laboratory of Regenerative Biology, Guangzhou Institutes of Biomedicine and Health, Chinese Academy of Sciences, Guangzhou 510530, P. R. China; 3 Department of Biology, South University of Science and Technology of China, Shenzhen 518055, P. R. China; 4 Shenzhen Research Institute, The Chinese University of Hong Kong, Shenzhen 518057, P. R. China

## Abstract

Transcription activator-like effector nucleases (TALENs) are novel engineered DNA nucleases, and have been proven to be effective for gene specific targeting in various species. Recently we reported gene disruptions in *Xenopus* embryos by using TALENs. Here we summarize the protocol that is used in our studies for gene disruption. This protocol covers selection of TALEN targeting sites, TALEN assembly with a modified Golden Gate method, and injection of TALEN mRNAs into *Xenopus tropicalis* embryos. We also provide details for detection of somatic and germ line transmitted mutations. And finally, we briefly describe establishment of knockout *Xenopus* lines. This protocol will facilitate broader applications of TALENs in studies of *Xenopus* biology.

## Introduction

*Xenopus tropicalis* is an animal model widely used in studies for both vertebrate development and diseases because of various advantages including its vigorous fecundity, fast embryonic development, short generation period, similar morphogenetic movement as higher vertebrates and feasibility for forward genetics [1,2]. However, the barriers that impeded this model for broader use were lack of embryonic stem cell lines and the methodology of homologous recombination. Therefore it is difficult to perform specific gene targeting in *Xenopus tropicalis* with conventional reverse genetic methods. Such obstacles have been recently overcome by engineered endonuclease tools, the zinc finger nucleases (ZFNs) [3] and the transcription activator-like effector nucleases (TALENs) [4,5]. In particular, TALENs have been proven to be very effective for gene disruption in *Xenopus* as well as other animal models [5-15]. In this article, we will describe how to perform specific gene disruption with TALENs in *Xenopus tropicalis* embryos, and eventually establish knockout *Xenopus tropicalis* line.

## Transcription activator-like effector nucleases

**Figure 1:**
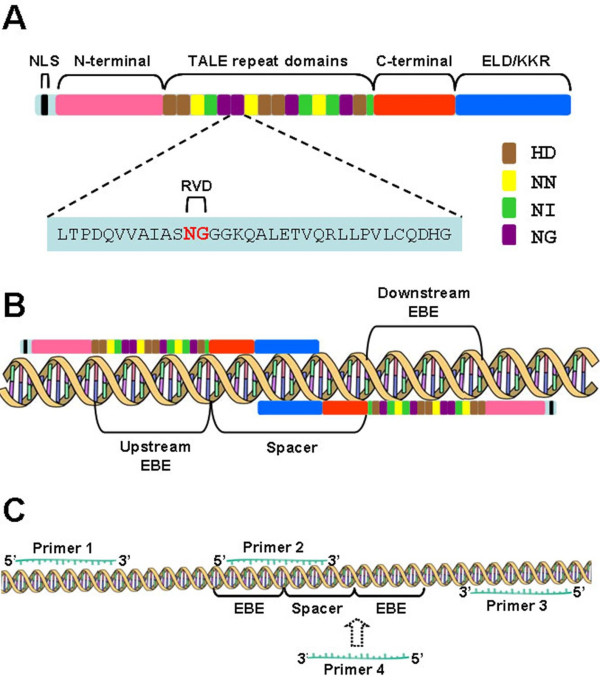
**Schematic diagrams of TALEN structure and PCR-based assay for detection of mutagenesis.** (**A**) Schematic drawing of TALEN structure. TALEN architecture was optimized for both zebrafish and *Xenopus*. Each TALEN monomer consists of a nuclear location signal (NLS), 152 amino acids deletion N-terminal, 63 amino acids from C-terminal, the TALE repeat domains, and modified Fok I nuclease domain ELD/KKR. Each TALE repeat unit consists of 34 amino acids, in which the amino acids at positions 12 and 13 are called ‘repeat-variable di-residues’ (RVDs). The RVD determine binding specificity to DNA bases following the code that NG, NI, HD, and NN respectively recognized thymine, adenine, cytosine and guanine. (**B**) Schematic diagram illustrating binding of TALENs to their targeted DNA sites. Each monomer of a TALEN pair recognizes and binds DNA via upstream or downstream EBE individually. Two Fok I nuclease domains ELD and KKR dimerize and function as endonuclease, generating DNA double strands break (DSB) at spacer between the two EBE sites. (**C**) PCR assay determining indel mutations induced by TALENs. The DNA fragment coving two EBE sites were amplified with Primer 1/3, the amplicons were then subcloned into pMD18-T using TA cloning. Primer 1/3 were employed to check insertion of targeted sequence after TA cloning. Primer 2 covered the joint region between upstream EBE and the spacer. When indel mutations were amplified in the spacer region, no amplicons would be generated by Primer 2/3. The corresponding plasmids were then sequenced to verify TALEN-induced mutations. Primer 1/4 may also be introduced to this PCR assay for detection of some mutations close to the downstream EBE site, which could not be captured by Primer 2/3. Not drawn to scale.

TALENs are novel engineered nucleases for gene disruption and have been successfully applied in a variety of organisms and cell types including plant [14], *Drosophila*[10], *C. elegans*[13], zebrafish [9,11], *Xenopus*[4,5], rat [12], livestock [6], human somatic cells [7], and human pluripotent stem cells [8]. TALENs consist of two distinct domains, an engineered DNA-binding domain derived from the transcription activator-like effector (TALE) that provides the capability and specificity for binding target DNA sequence [16,17], and a non-specific restriction endonuclease Fok I domain fused to the C-terminal of TALE that confers the nuclease activity of TALENs [18] (Figure 1A).

TALEs are proteins secreted by *Xanthomonas* plant bacteria. These proteins bind to promoters of their target genes in host cells and activate expression of plant genes in favor bacterial infection [16,17]. A TALE protein typically consists of a translocation N-terminal domain, a nuclear localization signal, C-terminal domain, and a central repeat domain. The central repeat domain indeed provides DNA binding specificity of a TALE protein. Each repeat unit consists of 34 amino acids, and their sequences are almost identical except for two amino acids at the 12th and 13th positions. These two amino acids which determine the binding specificity of TALE proteins to DNA bases are called ‘repeat-variable di-residues’ (RVDs). NI, NG, HD and NN are known to preferentially recognize adenine (A), thymine (T), cytosine (C), and guanine (G)/adenine (A), respectively (Figure 1A). Following this code, a given DNA sequence can theoretically be recognized by an assembled array of central repeats of TALE proteins.

Effective endonuclease activity of TALEN requires dimerization of two monomers that recognize individual DNA target sites. The Fok I domains on the two monomers designed to bind to adjacent DNA target sites will form a dimer, in turn become activated, generating DNA double-strand break (DSB) at the spacer region between two EBEs (Figure 1B). So far, no clear evidence indicates that TALEN binding to its target sequence is context dependent, but natural TALE binding site always begins with a thymine. Moreover, the off-target effects of TALENs have not been well studied yet, and should be addressed in detail by research community in the future.

## TALEN EBE selection and Golden Gate assembly

As mentioned above, TALENs are effective tools for gene disruption; the key point for the application of TALENs is fast and efficient assembly. Currently, there are various methods for TALEN assembly, such as Golden Gate assembly [7], PCR-based modular assembly [19], FLASH assembly [20], as well as some commercially available approaches including GeneArt® Precision TALs (Life Technologies) and TALEN™ (Cellectis bioresearch). Among them, Golden Gate assembly has been proven to be easy, fast and effective, therefore suitable for common laboratories. We chose Golden Gate strategy for TALEN assembly in our studies. The principle of Golden Gate assembly is based on type IIS restriction enzymes that can cleave DNA out of their binding site [21]. In addition to the original report [7], we made two constructs, pCS2-TALEN-ELD/KKR, which are feasible for microinjection in both zebrafish and *Xenopus* embryos. We shortened both N- and C-terminal of TALE backbone, and replaced wild type Fok I with ELD/KKR. ELD and KKR are both derived from the wild type Fok I and can form a heterodimer to reduce potential off-target effects [22]. Our modified Golden Gate TALEN system has been proven to be suitable and effective in *Xenopus* embryos [5].

Previous reports indicated natural TALE binding site always begins with a thymine and its central repeat domain ends with a half repeat unit (0.5 repeat) [16,17]. In line with this view, the crystal structure of TALE also suggests that thymine is necessary for its recognition and binding to DNA, although few reports showed TALE could still bind to its target sequence without the thymine [23]. The TALE architecture we chose is Δ152N-RVDs- + 63C. It has been successfully used for gene disruptions [24-26], and for genome editing in zebrafish embryos more recently [27]. The length of EBE and the spacer were set differently in various studies [7,24,26,28]. We routinely assemble TALENs harboring 15-20 repeats as such setting showed good DNA binding specificity and DNA disruption efficiency in *Xenopus* embryos [5]. The spacer between two EBE sites is 14-17 bps in our TALEN system.

In this TALEN mutagenesis protocol, we always select TALEN EBEs that follow a thymine. The RVDs of HD, NI, and NG preferentially bind to specific nucleotide C, A and T respectively, but NN is able to recognize both G and A [24]. Other RVD NK and NH recently were reported to have higher binding specificity to G, however, TALE reporters harboring either NK or NH showed less activity than NN [29,30]. Therefore currently NN is still the most commonly used RVD for recognizing G. In our studies, we tried to avoid G on the target DNA sequence in order to reduce potential off-target effects caused by the ambiguity of NN. There are also two web-based programs public available (http://taleffectors.com/tools, and https://tale-nt.cac.cornell.edu/node/add/talen) for TALEN EBE selection.

## DNA Repair after double strand break induced by TALENs

After TALE target selection, we assemble TALE repeats into pCS2-TALEN-ELD/KKR vectors. TALENs with wide type Fok I can form homodimers that may cause off-target effects. ELD/KKR are modified Fok I nuclease domains each of which carries three point mutations. TALENs harboring ELD and KKR were proven to have less off-target effects and higher efficiency because of the obligatory heterodimer formation [22]. Upon dimerization of ELD and KKR, a pair of TALENs that binds to adjacent EBEs at a locus of interest is expected to cleave DNA double-strand at the spacer, generating DNA double-strand break. The DSB will activate DNA repair mechanism of either non-homologous end joining (NHEJ), or homologous recombination (HR) if the proper DNA templates exist [18]. The repair of DSB by NHEJ results in small insertions or deletions (indels) at the cleaved site, leading to disruption of gene function. Repair of DSBs by HR allows integration of exogenous sequences at target site.

## Microinjection of TALEN mRNAs into *Xenopus* embryos

The protocol for TALEN assembly will be described in the section of Experimental procedure. After we obtain TALEN constructs that target a DNA sequence of interest, these two TALEN plasmids will be linearized by NotI (NEB) and the mRNAs for microinjection will be transcribed *in vitro* using the linearized DNA as templates (Ambion). After purification, quality and integrity of the synthesized mRNAs will be checked by denature agarose gel, and concentrations will be determined by spectrophotometer. We normally take 500 pg mRNA/embryo as a trial dose for injection. The ovulation of female frogs, *in vitro* fertilization, embryo manipulation and microinjection were described below. Alternatively one can follow the protocol in the Harland *Xenopus tropicalis* website (http://tropicalis.berkeley.edu/home/).

## Evaluation of mutagenesis rate induced by TALENs

The somatic mutations in the injected embryos are examined at 48 hours after injection. Five injected embryos were randomly pooled and the genomic DNA was extracted for further PCR assay [5], and usually four embryo pools were used for this assay. To access the frequency of NHEJ-induced mutagenesis at the target sites, two pairs of primers were employed for this assay (Figure 1C). Primer 1/3 can bridge the entire two EBE regions, and primer 2/3 can link the spacer region and the downstream EBE region. PCR is performed with primers 1/3 using the extracted genomic DNA as template. The amplicons will be cloned into vector pMD18-T (Takara) or other equivalent vectors by TA cloning, colonies will be examined by colony PCR using primer pairs 1/3 and 2/3, respectively. In case primer 1/3 generate PCR fragment, but primer 2/3 fail to do so, it will suggest that the colony harbors mutation, and such colonies will be further examined by DNA sequencing. Mutagenesis rate is defined as the ratio of mutant colonies to total colonies [5].

$$\mathrm{The}\;\mathrm{mutation}\;\mathrm{rate}=\frac{\mathrm{Number}\;\mathrm{of}\;\mathrm{mutational}\;\mathrm{colonies}}{\mathrm{Number}\;\mathrm{of}\;\mathrm{total}\;\mathrm{positive}\;\mathrm{colonies}}$$

It should be pointed out that high-dose of TALEN injection may cause defects during embryonic development, revealed by curled body axis or microcephaly. To assess the possible toxicity of TALENs, we also calculate the ratio of malformation and dead embryos at 48 hr after microinjection. Such defective embryos will normally die around metamorphosis.

## Evaluation of germ line transmission

**Figure 2:**
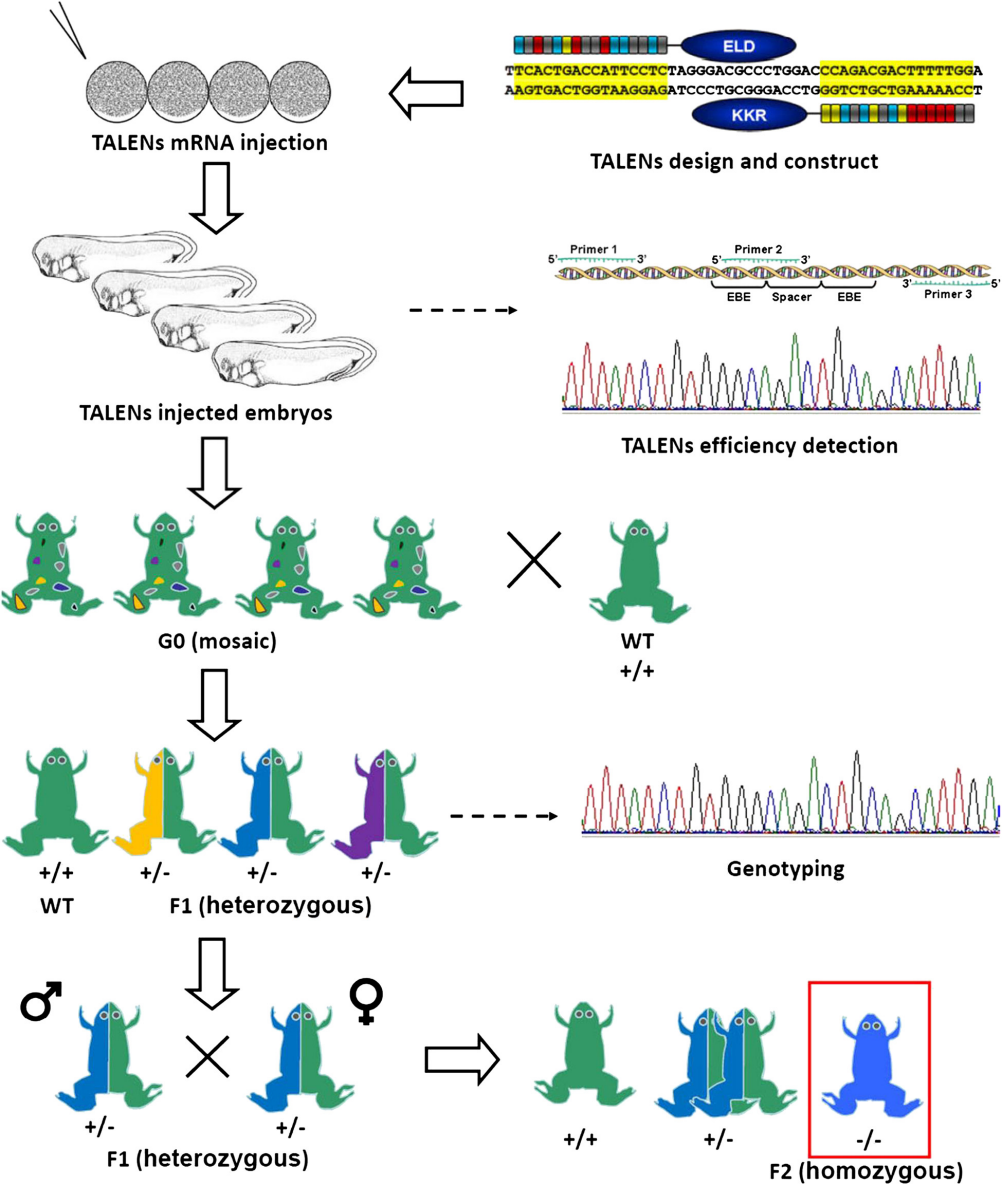
**Workflow for establishing gene knockout *Xenopus *lines by TALENs.** After selection of TALENs target site and assembly of TALEN plasmids, mRNAs encoding a pair of TALENs were microinjected into *Xenopus* embryos at one cell stage. The gene disruption efficiency was examined by PCR-based assay and afterwards sequencing. Some of the injected embryos were raised till sexual maturity. Individual G0 founder frog was mated with WT frog for germ line transmission to get F1 frogs*.* The F1 frogs were genotyped before metamorphosis by cutting tail and were raised to adulthood for intercross. Theoretically, ~25% offspring from F1 *Xenopus* will be homozygotes frogs carrying a specific gene mutation.

The germ line transmission is essential for establishment of a gene knockout frog line. In addition to evaluation of somatic mutations, the rest of injected embryos will be cultured to adult frog for examining germ line transmission of mutations. The G0 frogs are mosaic, carrying various mutations in their somatic cells. However, this situation normally will not affect the germ line transmission as individual sperm or oocyte only carries one mutation. To carry out germ line transmission of mutations, individual G0 frog will mate with wild type frog after HCG stimulation. The fertilized embryos (F1 generation) will be collected and cultured to stage 41 (about 48 hr post fertilization). Genomic DNA will be extracted from individual embryos and analyzed by PCR assay described above for accessing mutagenesis rate. If a F1 embryo carries mutation, it should be heterozygous, and theoretically approximate 50% colonies will harbor mutations. The mutagenesis positive colonies will be sequenced directly to confirm sequence alternations. The ratio for germ line transmission is defined as the number of mutated F1 embryos to the total examined F1 embryos. Sibling F1 embryos will be raised to sexual maturity. The genotyping for individual F1 frogs will be performed by PCR and afterwards sequencing using genomic DNA extracted from its tail before metamorphosis (Figure 2). We often find that several F1 embryos carry the same mutation. In that case, these two frogs will be selected for mating and the fertilized embryos (F2) will be collected. In theory, 25% offspring are homozygous at the target loci. The F2 frogs will be raised and genotyped for population expansion of this knockout frog line.

## Experimental procedure

Extraction and normalization of TALEN assembly vectors.

1. Recovery and extraction of TALE vectors. Obtain the Golden Gate vectors from Addgene (Cat #1000000016 or #1000000024). Use a sterile pipette tip or inoculation loop to dip bacteria from the bacterial stab and streak bacteria onto LB Agar plates with proper antibiotics (see Reagents). On the second day, pick a single colony and culture it in LB Broth with appropriate antibiotics overnight. Extract vector DNA using a standard miniprep kit (Favorgen)^a^, and adjust DNA concentration to 150 ng/μL with TE buffer. Store plasmids DNA at −20°C.

2. EBE sequence selection. We routinely choose TALEN targeting sequences by the following criteria: 1) Thymine (T) is at position 0 and the EBE sequence follows this T; 2) the spacer sequence is around 14-17 bp; 3) minimize the number of guanine (G) residues in EBE sequences to reduce ambiguity of RVD NN; 4) select EBEs in an exon or a junction region between intron and exon.

TALENs Assembly with Golden Gate Method.

**Figure 3:**
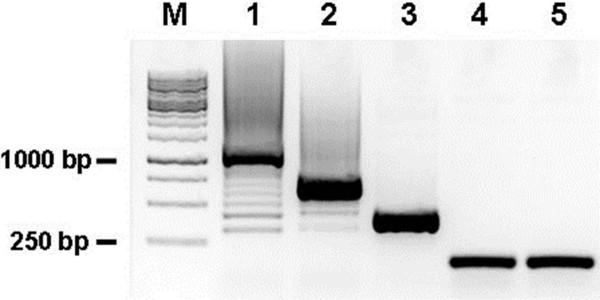
**Agarose gel image showing PCR amplicons from different steps of Golden Gate assembly.** pFUS_A vectors contain 1st-10th RVDs repeats (Lane 1) and pFUS_B5 vectors harbor 11th-15th RVDs repeats (Lane 2) in Golden Gate assembly round 1. The smeared ladder bands starting at ~300 bp with increase of approximate every 100 bp indicated successful RVD assembly and ligation to pFUS_A/B. Unsuccessful insertion led to incorrect size of amplicons (Lane 3). Lane 4 and 5 represent colony PCRs for pCS2-TALEN-ELD/KKR vectors carrying RVDs in Golden Gate assembly round 2. Correct PCR fragments amplified by primer pair of CTalF/CTalR are about 200 bp after assembly of the RVDs repeats from pFUS_A, pFUS_B5, and final half RVD repeat, into the pCS2-TALEN-ELD/KKR vectors.

3. Assemble TALENs with Golden Gate method. The TALEN assembly in principle follows the method published elsewhere [7] except that we made two vectors pCS2-TALEN-ELD/KKR for the last step of assembly [5]. We also have optimized some reaction conditions. If the TALE length is 12-21, pick vectors for RVD 1-10 and pFUS_A, then pick RVD vector 11 up to N-1 and pFUS_B#N-1. If the TALE length is 22-31, pick vectors for RVD 1-10 and pFUS_A30A, RVD vector 11-20 and pFUS_A30B, then pick vectors 21 up to N-1 and pFUS_B#N-1. The last RVD is half repeat and will be assembled in the cloning round 2.

4. Set up Golden Gate reaction mix (Cloning Round 1) listed in Table 1.

5. Perform ligation on a thermal cycler from step 4 using the following program in Table 2.

6. Treat the ligation mix with Plasmid-Safe nuclease. Add 0.5 μL ATP (25 mM) and 0.5 μL Plasmid-Safe™ ATP-Dependent DNase (Epicentre) to each reaction mix. Incubate at 37°C for 1 hour. Plasmid-Safe nuclease can digest all unligated linear DNA including incomplete ligation products with uncompleted repeats and remaining linearized RVD vectors and pFUS vectors.

7. Transform 50 μL competent cells (DH5α, Invitrogen) with ligation mix. Plate the transformed competent cells on Spectinomycin ^+^ LB Agar plate at 37°C overnight.

8. After 14-16 hr incubation, pick 7 pFUS_A colonies and 3 pFUS_B colonies. Resuspend each colony with 40 μL dH_2_O by vortex as PCR template. Prepare PCR mix as follows in Table 3. (The ligation efficiencies of both pFUS vectors are usually quite high. One can normally obtain positive candidate from 7 pFUS_A and 3 pFUS_B colonies).

9. Check positive clones by colony PCR with Golden Gate round 1 primers, pCR8_F & pCR8_R (see Materials) using the following cycling program in Table 4.

10. Check the amplicons with 1% agarose gel. If the ligation is successful, colony PCR will generate smearing ladder bands and the largest band is about 1.2 kb for pFUS_A vectors within 10 RVDs (Figure 3, Lane 1). The gel result of pFUS_B vector will also form smearing ladder bands with smaller size. The expected size was depended on the number of RVDs into pFUS_B vector (Figure 3, Lane 2).

11. Amplify the colonies harboring correct inserts in Spectinomycin ^+^ LB Broth by vigorous shaking at 37°C overnight.

12. Spin down the bacterial culture and perform mini prep to extract plasmid DNA (Favorgen). Adjust plasmid concentration to 150 ng/μL.

13. Set up Golden Gate reaction mix (Cloning Round 2) listed in Table 5.

14. Incubate the reaction mix at 55°C for 30 min (BsmbI digestion). Add 0.5 μL ATP (25 mM) and 0.5 μL T4 Ligase (NEB) to each reaction mix.

15. Perform ligation on a thermal cycler from step 14 using following program in Table 6.

16. Transform 50 μL competent cells (DH5α, Invitrogen) with ligation mix. Plate the transformed *E.coli* on Ampicillin ^+^ LB Agar plate at 37°C overnight.

17. After 14–16 hr incubation, pick 3 colonies from the selective agar plates and assess ligation (Step 13–15) by colony PCR (Step 8) using Golden Gate round 2 primers, CTalF & CTalR (see Materials), with following conditions Table in 7.

18. Examine the PCR products with 1% agarose gel. The expected size of amplicons should be about 200 bp (Figure 3, Lane 4 and 5).

19. Amplify the colonies that give correct bands in Ampicillin ^+^ LB Broth at 37°C overnight.

20. Perform mini prep to extract DNA of pCS2-TALEN-ELD/KKR plasmids containing full RVDs (Favorgen). Sequence the two constructs with two primers, NTalF & CTalR (see Materials) to confirm successful assembly.

**Table 1:** Ligation mix in Golden Gate cloning round 1

**Components**	**pFUS_A Mix (μL)**	**pFUS_B Mix (μL)**
Buffer 4 (NEB)	1	1
T4 Ligase (NEB)	0.8	0.8
BsaI (NEB)	0.8	0.8
RVD vectors (150 ng/μL)	0.6 × 10	0.6 × #N-1
pFUS_A/B vectors (150 ng/μL)	1	1
ATP (25 mM)	0.4	0.4
dH_2_O	0	6-(0.6 × #N-1)
Total	10	10

**Table 2:** Ligation program in Golden Gate cloning round 1

**Cycle number**	**Temperature (°C)**	**Time (min)**
1-15	37	5
	16	10
16	16	15
17	50	10
18	80	5

**Table 3:** Colony PCR mix in Golden Gate cloning round 1

**Components**	**PCR Mix (μL)**
5 × GoTaq Buffer	5
25 mM MgCl_2_	2.5
10 mM dNTP Mix (Fermentas)	0.5
Primer Fw (10 mM)	0.5
Primer Re (10 mM)	0.5
Colony suspension	5
GoTaq DNA polymerase (Promega)	0.5
dH_2_O	10.5
Total	25

**Table 4:** Colony PCR program in Golden Gate cloning round 1

**Cycle number**	**Temperature (°C)**	**Time (min)**
1	95	5
2-31	94	0.5
	52	0.5
	72	1.5
32	72	10
33	4	~

**Table 5:** Ligation mix in Golden Gate cloning round 2

**Components**	**pCS2-TALEN Mix (μL)**
Buffer 3 (NEB)	1
pFUS_A-RVDs vectors (150 ng/μL)	0.8
pFUS_B-RVDs vectors (150 ng/μL)	0.8
Last Repeats vectors (150 ng/μL)	0.6
BsmbI (NEB)	0.8
pCS2-TALEN-ELD/KKR vectors (150 ng/μL)	1
dH_2_O	5
Total	10

**Table 6:** Ligation program in Golden Gate cloning round 2

**Cycle number**	**Temperature (°C)**	**Time (min)**
1-3	16	10
	37	20
4	16	20
5	55	20
6	80	5

**Table 7:** Colony PCR program in Golden Gate cloning round 2

**Cycle number**	**Temperature (°C)**	**Time (min)**
1	95	5
2-31	94	0.5
	52	0.5
	72	0.5
32	72	10
33	4	~

Synthesize TALEN mRNAs *in vitro*.

21. After sequence confirmation of assembled TALEN constructs, linearize the pCS2-TALEN-ELD/KKR constructs containing RVD with NotI (NEB). Purify the linearized DNA with Gel/PCR purification kit (Favorgen)^b^ according to the manufacturer’s instructions.

22. Synthesize mRNAs for microinjection with mMessage mMachine SP6 kit (Ambion) using the linearized DNA (step 21) as templates. Purify the synthesized mRNA with RNeasy MiniElut Cleanup Kit (QIAgen). Check quality and integrity of the mRNA with denature agarose gel and measure RNA concentration with spectrophotometer.

Microinjection of TALEN mRNAs into *Xenopus* embryos and evaluation of somatic gene disruption efficiency.

23. Prior to mating, induce ovulation of female frogs with 20 IU human chorionic gonadotropin (hCG) at night before *in vitro* fertilizations. Inject another dose of 200 IU hCG on the next day. The female frogs lay eggs from 2 hr to 6 hr after hCG injection.

24. Dissect testes from a male frog, and cut testes of male frog into very small pieces and transfer them into 1xMMR (high concentration to keep sperm activity) on ice. Squeeze the ovulated female frog and collect eggs onto a petrdish. Pipet sperm mix onto eggs. Briefly mix them well and wait for 3-5 minutes. Pour 0.1xMMR into the dish to cover eggs till completion of fertilization.

25. Keep eggs at about 25°C throughout fertilization periods. Collect embryos and remove jelly coat from the eggs using 3% cysteine hydrochloride (in water, pH 7.8-8.0) after step 24.

Or follow the Harland tropicalis website to perform female ovulation, *in vitro* fertilization, and manipulation. (http://tropicalis.berkeley.edu/home/obtaining_embryos/hcg/hCG.html).

**Figure 4:**
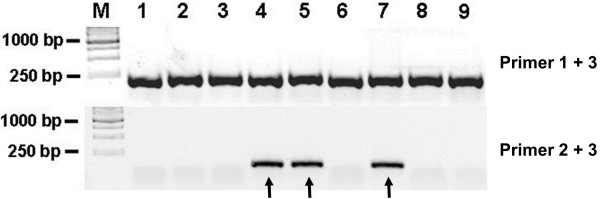
**Colony PCR results for evaluation of gene disruption.** Agarose gel showing an example of colony PCR to detect mutagenesis in ets1-TALEN injected *Xenopus tropicalis* embryos. The bands amplified by Primer 1/3 in upper panel (~200 bp) indicate the colonies harbor the TALEN targeted sites. PCR assay was also carried out with Primer 2/3 in lower panel. The colonies that can give upper bands but fail to give lower bands were the positive colonies carrying potential mutants. The colonies giving both upper and lower bands did not carry mutants (arrows indicated). Mutations were further confirmed by sequencing.

26. Microinjection is performed at about 22°C. After TALENs injection, culture the injected embryos in 0.1xMBS culture medium at 22°C overnight and then transfer them to 25°C. When the injected embryos reached about stage 41 (about 48 hr post fertilization), randomly collect and pool five TALEN-injected embryos, and extract genomic DNA with DNeasy blood & tissue Kit (QIAgen) from embryo pools according to the manufacturer’s instructions. Raise the rest of injected embryos to adult frogs for future germ line transmission.

27. Set up a PCR reaction to amplify the genomic region that covers two TALEN EBE sites. A pair of primers flanking the two EBE sites is employed for this PCR reaction (Figure 1C). According to our experience, we recommend the length of amplicons is less than 1 kb. Prepare PCR mix as following in Table 8.

28. After PCR reaction, check the size of PCR products, and recover the DNA with gel extraction kit (Favorgen). The purified amplicons are then cloned into pMD-18 T vector by TA cloning (Takara) using the following set-up in Table 9.

29. Incubate the ligation mix at 16°C for 60 min.

30. Transform 50 μL competent cells (DH5α, Invitrogen) with 10 μL ligation mix. Plate the transformed DH5α on Ampicillin ^+^ LB agar plate at 37°C overnight.

31. Randomly pick up at least 20 colonies for each embryo pool. Colony PCR is used to access mutagenesis rate at the targeted sites, using primer pair 1/3, and primer pair 2/3, respectively (Figure 4). If mutations are generated in the spacer region, no amplicon can be amplified using the primer pair 2/3. Verify mutation by direct sequencing.

32. Raise the sibling TALENs injected *Xenopus* embryos till sexual maturity according to (http://tropicalis.berkeley.edu/home/husbandry/raisetads.html) or protocol published elsewhere [31]. It takes about 5 months for the injected embryos to reach sexual maturity, and longer period is also expected depending on the feeding conditions. Pigment patch appearing in frog’s claws suggest maturity of male froglets.

33. When TALENs targeted frogs are ready for germ line transmission, mate a male or female G0 frog with a wild type frog. The details are described as follows: (http://tropicalis.berkeley.edu/home/obtaining_embryos/natural_mating.html). Briefly prior to mating, inject both male and female frogs with 20 IU hCG at the night before natural mating and keep separately to avoid amplexus and possible fertilizations. Inject another dose of 200 IU hCG to boost reaction of both male and female frogs and place them together without disturbances on the next day.

34. Usually female frogs lay eggs from 2 hr to 6 hr after the second hCG injection. Keep frogs at about 25°C throughout the priming and natural mating periods [31].

35. Collect embryos and remove jelly coat from the eggs using 3% cysteine hydrochloride (in water, pH 7.8-8.0). Culture the embryos in 0.1×MBS at 25°C until at stage 41. Randomly collect 20 embryos, extract genomic DNA from single embryo, and leave the sibling embryos to develop to adult frogs.

36. Use the method described in step 27–31 to determine whether the F1 embryos carry mutations, and calculate the rate for germ line transmission. (number of embryos harboring mutations/total number of examined embryos). After the sibling embryos developed till stage 45, cut tail for DNA extraction to determine the genotype at the targeted loci. Select the larvae carrying open reading frame (ORF)-shift mutations and raise them to sexual maturity for the intercross^c^.

37. After population expansion of selected F1 adult frogs, mate the F1 frogs. About 25% offspring from intercross of F1 adult frogs are homozygous at the targeted loci according to Mendel’s law. Expand the frog population with this genome type, and maintain this *Xenopus* mutant line.

**Table 8:** PCR mix for amplification of target genomic DNA

**Components**	**PCR Mix (μL)**
5 × GoTaq Buffer	5
25 mM MgCl_2_	2.5
10 mM dNTP Mix (Fermentas)	0.5
Primer 1/2 (10 mM)	0.5
Primer 3 (10 mM)	0.5
Genomic DNA	0.5
GoTaq DNA polymerase (Promega)	0.5
dH_2_O	15
Total	25

**Table 9:** TA cloning mix

**Components**	**Ligation Mix (μL)**
pMD18-T Vector (Takara)	1
Insert DNA (~0.3 pmol)	4
Solution I	5
Total	10

## Conclusion remarks

In our previous studies, TALENs exhibited remarkable efficiency of gene disruption as well as broad targeting spectrum of DNA sequence in genome [5]. Those advantages make TALENs a promising gene disruption approach for specific gene targeting in various animal models including *Xenopus*. In this article, we described the TALENs protocol currently used in our laboratory for generating mutations in *Xenopus tropicalis* embryos. We listed the criteria for EBE selection and the protocol for TALENs assembling. The methods of embryo manipulation and microinjection are basically the same as those used in other laboratories. We also described a PCR-based method for detecting the targeted gene disruption efficiency and procedures for establishing a gene-specific knockout *Xenopus* line from TALENs injected embryos. The PCR-based evaluation method is simple and reliable. It may miss some mutations that close to the downstream EBE site. We suggest to introduce a forth primer (Figure 1C) in order to capture all the possible mutations. TALENs display high efficiency for generating somatic mutations in G0 embryos. However it is worth noting that whether TALEN can be used the same as antisense morpholino oligonucleotides cannot be concluded before conducting comparable studies systematically. *ptf1a/p48* TALEN can induce complete loss of *xpdip* expression as did *ptf1a/p48* morpholino [5], but it seems that the efficiency is not as profound as that with *ptf1a/p48* morpholino knockdown in *Xenopus laevis* published previously [32]. We have observed similar effects with other genes. One explanation could be that the TALEN-injected embryos are mosaic and the gene disruption efficiencies vary in different embryos. And even in one embryo harboring mutations, the somatic mutations are different [5,33]. Moreover, TALEN cannot block functions from the maternal mRNAs. Such complexity may make it difficult to interpret phenotype induced by TALENs in G0 embryos. Therefore, we suggest that morpholinos cannot be replaced by TALENs currently. Although our protocol is for gene disruption in *Xenopus* embryos, it is also feasible for zebrafish with minor modifications of the setting for microinjection. With rapid growth of research on TALENs, accumulating data indicate that TALEN is an easy but robust tool for reverse genetics study. Engineered TALENs will facilitate and expand applications in various organisms for future research.

## Materials

Primer List (Table 10).

**Table 10:** Primers for Golden Gate assembly and sequencing

**Primer name**	**Sequence**
pCR8_F	TTGATGCCTGGCAGTTCCCT
pCR8_R	CGAACCGAACAGGCTTATGT
CTalF	CGTTGACCAACGACCACCTC
CTalR	CTAGTTGGGATCCGGCAAC
NTalF	GATGACAAGGGTACCGTG

Golden Gate vectors (Addgene Cat #1000000016 or #1000000024) (Table 11).

**Table 11:** Vectors in Golden Gate Kit (This kit can be purchased from Addgene)


**Tetracycline**									
pNI1	pNI2	pNI3	pNI4	pNI5	pNI6	pNI7	pNI8	pNI9	pNI10
pHD1	pHD2	pHD3	pHD4	pHD5	pHD6	pHD7	pHD8	pHD9	pHD10
pNN1	pNN2	pNN3	pNN4	pNN5	pNN6	pNN7	pNN8	pNN9	pNN10
pNG1	pNG2	pNG3	pNG4	pNG5	pNG6	pNG7	pNG8	pNG9	pNG10
**Spectinomycin**									
pLR-NI		pLR-HD		pLR-NN		pLR-NG			
pFUS_A		pFUS_A30A		pFUS_A30B					
pFUS_B1		pFUS_B2		pFUS_B3		pFUS_B4		pFUS_B5	
pFUS_B6		pFUS_B7		pFUS_B8		pFUS_B9		pFUS_B10	

TALENs backbone vectors (Ampicillin)

pCS2-TALEN-ELD, pCS2-TALEN-KKR.

### Reagents

T4 DNA ligase (NEB, Cat. no. M0202M)

NotI (NEB, Cat. no. R0189L)

BsaI (NEB, Cat. no. R0535L)

BsmbI (NEB, Cat. no. R0580L)

Plasmid-Safe™ ATP-Dependent DNase (Epicentre, Cat. no. E3110K)

GoTaq DNA polymerase (Promega, Cat. no. M8295)

pMD18-T vector (Takara, Cat. no. D101A)

FavorPrep Plasmid DNA Extraction mini Kit (Favorgen, Cat. no. FAPDE001-1)

DNeasy blood&tissue Kit (QIAgen, Cat. no. 69504)

GEL/PCR Purification Kit (Favorgen, Cat. no. FAGCK001-1)

mMessage mMachine SP6 Kit (Ambion, Cat. no. AM1340)

RNeasy MiniElute Cleanup Kit (QIAgen, Cat. no. 74204)

LB Broth medium (Usb, Cat. no. 75852)

LB Agar medium (Invitrogen, Cat. no. 22700–025)

Amplicillin (Sigma, Cat. no. A1593)

Spectinomycin (Sigma, Cat. no. S4014)

Tetracycline (Sigma, Cat. no. 87128)

GeneRuler 1 kb DNA Ladder (Fermentas, Cat. no. SM0311/2/3)

DH5α (Invitrogen, Cat. no. 18263–012)

## Endnotes

^a^ Miniprep can be performed by equivalent commercial kit.

^b^ Gel extraction or PCR purification can be performed by equivalent commercial kit.

^c^ It is critical to perform genome typing at the TALENs targeting sites for all adult F1 frogs prior to mating. If we find that a few male and female F1 frogs carry identical mutation, we will mate them directly. If only one frog has a particular genome type, we cross this frog with wild type frog again to expand the population.

### Ethical approval

All animal experiments were performed following approval from the University Animal Experimentation Ethics Committee (AEEC) of The Chinese University of Hong Kong.

## Competing interests

The authors declare that they have no competing interests.

## Authors’ contributions

YL, HZ, YLC, XGG, and YD wrote this methodology paper and revised it. All authors read and approved the final manuscript.
